# Nanopaper Properties and Adhesive Performance of Microfibrillated Cellulose from Different (Ligno-)Cellulosic Raw Materials

**DOI:** 10.3390/polym9080326

**Published:** 2017-07-31

**Authors:** Stefan Pinkl, Stefan Veigel, Jérôme Colson, Wolfgang Gindl-Altmutter

**Affiliations:** 1 Competence Centre for Wood Composites and Wood Chemistry, Wood K Plus, Linz 4040, Austria; wolfgang.gindl-altmutter@boku.ac.at; 2 Department of Materials Science and Process Engineering, BOKU—University of Natural Resources and Life Sciences, Vienna 1180, Austria; stefan.veigel@boku.ac.at (S.V.); jerome.colson@boku.ac.at (J.C.)

**Keywords:** nanocellulose, sugar beet, wood, nanopaper, bio-adhesive

## Abstract

The self-adhesive potential of nanocellulose from aqueous cellulosic suspensions is of interest with regard to a potential replacement of synthetic adhesives. In order to evaluate the performance of microfibrillated cellulose from different (ligno-)cellulosic raw materials for this purpose, softwood and hardwood powder were fibrillated and compared to sugar beet pulp as a representative non-wood cellulose resource, and conventional microfibrillated cellulose produced from bleached pulp. An alkali pre-treatment of woody and sugar beet raw materials enhanced the degree of fibrillation achieved, same as TEMPO-mediated oxidation of microfibrillated cellulose. Nanopapers produced from fibrillated material showed highly variable density and mechanical performance, demonstrating that properties may be tuned by the choice of raw material. While nanopaper strength was highest for TEMPO-oxidated microfibrillated cellulose, fibrillated untreated sugar beet pulp showed the best adhesive performance. Different microscopic methods (AFM, SEM, light microscopy) examined the interface between wood and fibrillated material, showing particular distinctions to commercial adhesives. It is proposed that fibrillated material suspensions, which achieve bond strength up to 60% of commercial urea-formaldehyde adhesive, may provide a viable solution to bio-based adhesives in certain applications where wet-strength is not an issue.

## 1. Introduction

Adhesive bonding is a key processing step in the production of modern engineered materials. Recently, there is a growing interest in bio-based solutions for adhesive bonding as an alternative to well-established high-performance fossil-based adhesives. One approach consists of the synthesis and use of partially or fully bio-based adhesives [1]. Lignin is a widely available resource in this context and intense efforts are being undertaken towards the partial replacement of phenol in the synthesis of phenol-formaldehyde based resins [2]. Even so, the most important hindrance with regard to broad lignin utilization in adhesive synthesis is still its lack of reactivity compared to phenol [1]. Tannins as a bio-based adhesive component extracted from the bark or xylem of trees are polyhydroxyphenols which start auto condensation when hexamine is added. Particleboards with internal bond strength of 0.9 MPa are already reported [1,3,4,5].

A different approach of substituting fossil-based adhesives may be found in the use of intrinsic bonding forces for binderless bio-based materials, such as those realised in paper. Numerous examples for such approaches may be found in literature. Binderless wood fiberboards are achieved with the aid of self-bonding forces of lignin in well-established wet-processing technology, where decomposition products of hemicellulose, hydrogen bonding and molecular entanglement contribute to the formation of bonds [6,7,8,9,10,11]. Similarly, the thermal behavior of lignin at higher temperatures was proposed to be responsible for the bonding of high-density coconut husk boards [12]. Steam explosion is a useful pre-treatment in this context, as shown for softwood fiber [13,14], and particleboard from oil palm frond [15]. In addition to the activation of native lignin, technical lignin may be an additive providing adhesive bonding, as shown for sheets of steam-exploded Miscanthus sinensis. Resulting fiber boards show flexural strengths higher than 60 MPa and internal bond strengths of 3.5 MPa [16].

Carbohydrates are also an interesting additive enabling the manufacture of binderless boards. The addition of 20% glucose to oil palm biomass improved the internal bond strength of particleboards from 0.6 to 1.7 MPa [17]. Hemicellulose as a branched polymer with a lower molecular weight compared to cellulose also offers possibilities to develop self-adhesive forces. Hemicelluloses can be easily hydrolyzed into their constituting monosaccharides (pyranoses and hexoses) with acids and mild temperatures (100–130 °C). Hydrolysis of pyranose leads to furfural while hexoses degrade to 5-hydroxymethyl-2-furfuraldehyde, whereby both molecules are able to polymerize again [18,19]. In wood bonding, these molecules are able to react with other wood components e.g., lignin. From bagasse, lignocellulosic composites can be produced by in situ formation of self-adhesive forces [20]. The Thermodyn process from 1940 is also based on these self-adhesive forces [21].

Through their high specific surface area and hydrophilic surface, cellulose nanomaterials are also capable of providing adhesion. It is well known that the addition of microfibrillated cellulose to the papermaking process benefits paper strength [22,23]. Recently, several studies demonstrated the usefulness of wet microfibrillated cellulose for providing adhesive bonding in paper laminates [24] and in various wood-based composite board products [25]. A similar mechanism is at work in the Zelfo process, where partial fibrillation of plant material allows the production of novel, fully bio-based and binderless structural materials [26].

In the present study we will apply the concept of using fibrillated cellulosic material as a binder to a broad variety of fibrillated (ligno-)cellulosic materials. The hypothesis that fibrillated material can build-up substantial adhesive forces will be examined in the present paper together with the performance of nanopapers produced from these materials.

## 2. Materials and Methods

### 2.1. Preparation of Fibrillated Material

Dried sugar beet pulp obtained from a sugar factory in Tulln (Agrana Zucker GmbH, Tulln an der Donau, Austria) was milled in a cutting mill (SM1, Retsch GmbH, Haan, Germany), first to a particle size <2 mm and in a second run <0.2 mm. One variant was fibrillated without further pre-treatment, whereas a second variant was extracted in batches of 20 g with 200 mL NaOH (0.5 M) for 2 h at 80 °C while stirring. The supernatant was removed and the pulp was washed and centrifuged before extracting a second time with 100 mL NaOH (0.5 M) for 15 min at 20 °C while stirring. The washed residue was bleached with 10 g NaClO_2_ (80% Techn., Carl Roth GmbH & Co. KG, Essen, Germany) in an acetate buffer (pH = 4.9, prepared from 5 g acetic acid (100% p.a., Carl Roth GmbH & Co. KG), 5 g sodium acetate (≥98.5% pure, Carl Roth GmbH & Co. KG) and 25 mL deionized water) for 2 h at 70 °C while stirring. Then the suspension was centrifuged and washed until the chlorine smell disappeared [27].

For reference purposes, a broad variety of cellulosic sources was used for the production of fibrillated material. Fibrillated wood was prepared by disintegrating beech- and spruce wood boards into chips and subsequent milling as described above. While one variant of wood particles was further fibrillated without chemical pre-treatment, a second variant was extracted in batches of 10 g with 250 mL 5% (*w*/*v*) NaOH (≥99% p.a., Carl Roth GmbH & Co. KG) for 2 h at 20 °C while stirring. Thereafter the supernatant was decanted and wood particles were washed with deionized water to pH = 7. Conventional microfibrillated cellulose was produced from lignin-free never-dried beech sulphite pulp provided by Lenzing AG, Wien, Austria. While one variant of pulp was fibrillated as received, a second variant was treated by oxidation using the well-known TEMPO/NaClO/NaClO_2_ system [28,29] at 60 °C for 2 h.

All the variants described above were diluted to a solid content of 0.5% in water and dispersed using an Ultra Turrax device (IKA®-Werke GmbH & CO. KG, Staufen, Germany). Mechanical fibrillation was carried out by applying 20 passes through a laboratory homogenizer APV1000 (SPX FLOW Inc., Delavan, WI, USA) at a pressure of 70–80 MPa. An overview of sample codes used together with the most relevant treatments is given in Table 1.

### 2.2. Film Casting and Tensile Test

After homogenization, the suspensions (solid content 0.5%) were filled in petri dishes (1000 mL) and stored under a fume hood at room temperature for 48 h to evaporate water. Test specimens were cut with a razor blade punch in dog bone shape, with 25 mm test length. Outer parts were strengthened with an edging tape to prevent breaks in the clamping area. After one week storage at 20 °C and 65% RH, specimens were tested with a static materials testing machine (Z020, Zwick GmbH & Co. KG, Ulm, Germany). All samples were tested to failure with a constant test speed of 1 mm·min^−1^. Tested films were used for further microscopic studies.

### 2.3. Microscopy

Fibrillated suspensions were further diluted to 0.005% (*w*/*w*) with deionized water and dropped on conventional mica platelets glued on atomic force microscope (AFM) specimen discs. The AFM (Dimension Icon, Bruker, Bremen, Germany) was used in tapping mode with an Otespa tip (resonant frequency 300 kHz, spring constant 42 N·m^−1^). Overview pictures (20 µm × 20 µm, amplitude error picture) and zoom pictures (2 µm × 2 µm, Z sensor picture) were consulted for analysis. On zoom pictures, the height of the 10 smallest fibrils was measured to determine an average smallest fibril diameter. Therefore the height profile extraction function of an AFM visualization and analysis software (Gwyddion 2.44, Brno, Czech Republic) was applied.

Scanning electron microscope (SEM) (Quanta FEG 250, FEI, Brno-Černovice, Czech Republic) was used for pictures of fractured film surfaces after the tensile tests, operated with a 5 kV electron beam. Before scanning, surfaces were coated with gold vapour using a sputter coater (Scancoat Six, HHV Ltd., Crawley, UK) adjusting 1 mbar, 20 mA and 3–4 kV for 240 s.

### 2.4. Determination of Adhesive Bond Strength by Lap-Shear Testing

Homogenized suspensions were adjusted to a solid content of 3% (*w*/*w*) by careful drying in a drying chamber (Memmert UFB 500, Schwabach, Germany) and further used to glue spruce slats following the European standard EN 302-1:2013 [30]. Slats (length 500 mm × width 120 mm, annual ring alignment 30–90°) conditioned at 20 °C and 65% RH were planed to a final thickness of 5 mm shortly before gluing. Aliquots of suspensions corresponding to 30 g m^−2^ solid matter were spread with a spatula. At a specific pressure of 0.5 MPa and 50 °C the slats remained in a laboratory press (Langzauner GmbH, Lambrechten, Austria) for 72 h. 10 test specimens (length 150 mm × width 20 mm) were cut after a further storage at above climate conditions until constant weight. Testing of longitudinal tensile shear strength was performed in tensile test mode with 0.15 mm·min^−1^ deformation rate.

In a second experiment, the applied amount of fibrillated material was increased to 60 and 120 g m^−2^ solid matter. Therefore, the solid content of the suspensions was increased to 6.7% and 11.7% which is more or less the maximum applicable amount without squeezing out of the slats. Pressing parameters were adjusted to 120 °C and 1 h pressing time in order to account for increased moisture. Mean values were calculated for each group and compared by one-way analysis of variance (Post-hoc-test Tukey b, Levene’s test for equality of variances, Kolmogorov-Smirnov test for normal distribution, *p* ≤ 0.05).

## 3. Results and Discussion

### 3.1. Microfibrillated Cellulose from Different Raw Materials

**Figure 1:**
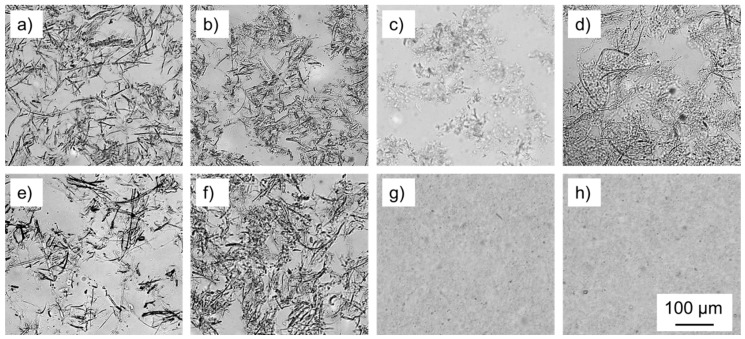
Light microscopic images of different fibril suspensions: (**a**) spruce; (**b**) beech; (**c**) sugar beet; (**d**) MFC; (**e**) NaOH spruce; (**f**) NaOH beech; (**g**) NaOH sugar beet; (**h**) TEMPO MFC.

The morphology of microfibrillated cellulose from different raw materials was studied by means of transmitted light microscopy in order to obtain an optical impression of the degree of fibrillation achieved. In addition, atomic force microscopy was used to obtain quantitative measurements of the smallest fibril diameters achieved. At first glance, light microscopy shows very clear differences between the different raw materials used (Figure 1).

With all samples, the beneficial effect of alkali pre-treatment or TEMPO oxidation, respectively, on the degree of fibrillation achieved is evident. Even so, the wood powders spruce and beech appear to be more recalcitrant towards fibrillation even after alkali pre-treatment, as numerous larger fiber fragments are prevalent (Figure 1e,f). This effect is more pronounced for NaOH spruce compared to NaOH beech. Compared to wood powders, sugar beet pulp provided small fibrils as far as this can be deduced from light microscopy (Figure 1c). While MFC (Figure 1d) still showed a high amount of fibrils discernible by light microscopy, this was no longer the case for NaOH sugar beet and TEMPO MFC, respectively, indicating very fine fibrils (Figure 1g,h).

**Figure 2:**
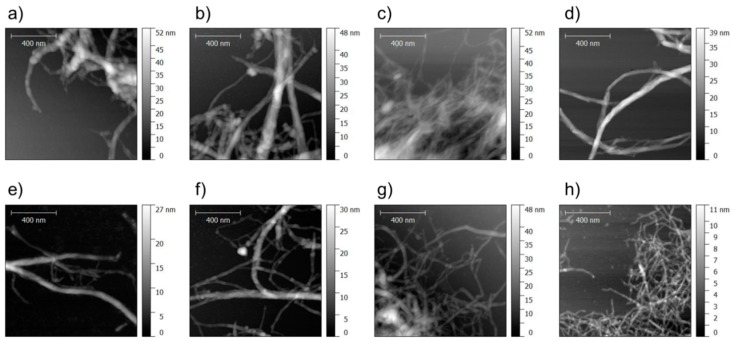
Atomic force microscopic (AFM) images of the smallest fibril fraction found in different fibril suspensions: (**a**) spruce; (**b**) beech; (**c**) sugar beet; (**d**) MFC; (**e**) NaOH spruce; (**f**) NaOH beech; (**g**) NaOH sugar beet; (**h**) TEMPO MFC.

**Figure 3:**
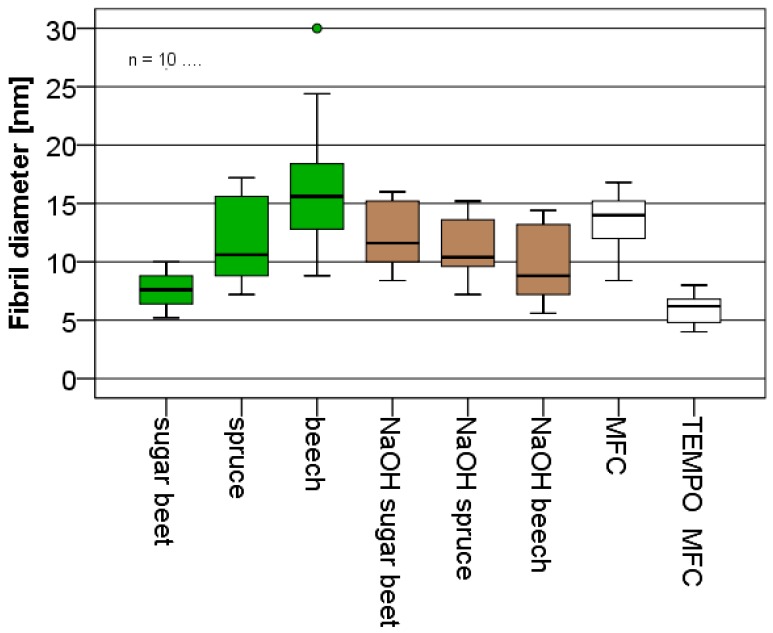
Box and whisker plot of the height of the smallest fibrils found in AFM topography images shown in Figure 2 (10 fibrils each were measured).

In general, it is difficult to capture the entire structural variability of (partly) fibrillated cellulose with one microscopic method alone. Therefore, AFM was used complementary to light microscopy in order to cover the smallest fibrillary structures found in each variant. It was assumed that the smallest fibril fraction is of particular relevance to the performance of MFC products, because even though its mass fraction may be small, its contribution to the overall accessible specific surface area is significant. In accordance with light microscopy (Figure 1), AFM (Figure 2) also reveals distinct differences in fibril fineness between the individual variants studied solely based on visual inspection. Quantification of fibril height directly from AFM images shown in Figure 3 confirms this visual impression. With regard to the average values of fibril height shown, it should be noted that these values represent only the lower tail of the distribution of fibril diameters and do not give an indication of characteristic overall fibril diameters.

### 3.2. Nanopapers Prepared from Different Fibrillated Materials

**Figure 4:**
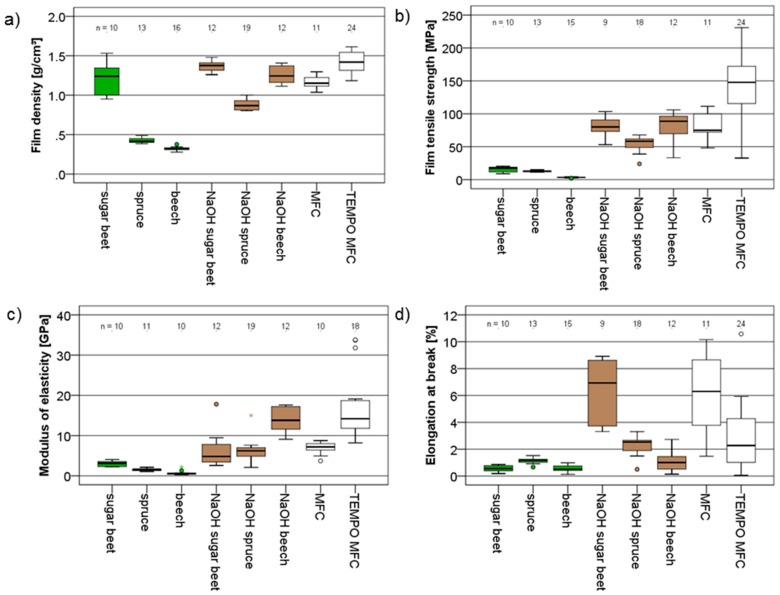
Box and whisker plots of (**a**) density; (**b**) tensile strength; (**c**) modulus of elasticity; and (**d**) elongation at break for nanopapers produced from different raw materials.

The tensile strength and modulus of elasticity of dry films of fibrillated cellulosic materials provide valuable insights into the different performance of fibrillated materials [31,32,33], which is why nanopapers were produced and tested prior to adhesion testing. Overall, a great diversity of film thickness, ranging between 0.02 mm and 0.15 mm and, concurrently, density ranging from 0.3 g·cm^−3^ up to 1.4 g·cm^−3^ was obtained (Figure 4a). The lowest film densities were observed for fibrillated untreated wood powders, showing that these materials were particularly recalcitrant towards proper fibrillation, whereas fibrillated sugar beet yielded high-density nanopaper even without pre-treatment. Improved fibrillation after alkali pre-treatment, which was already presumed based on the optical impression given by light microscopy (Figure 1), is confirmed by the consistent increase in nanopaper density observed for these variants. As for MFC, high density is observed, which is furthermore improved by TEMPO oxidation pre-treatment to the highest density values among all variants studied.

The mechanical performance (strength and modulus of elasticity, Figure 4b,c) of nanopapers in tensile testing shows trends similar to film density, with the notable exception of untreated fibrillated sugar beet. Even though high-density nanopaper with an average density of 1.25 g·cm^−3^ was obtained, the mechanical performance of this material is modest and very similar to untreated fibrillated wood powder. Overall, this group of three untreated fibrillated materials shows the lowest mechanical properties compared to all other variants. While this poor performance may be explained by a low degree of fibrillation together with a low film density in the case of untreated fibrillated spruce and beech, untreated sugar beet presumably performs poorly due to the presence of a substantial percentage of non-cellulosic constituents. Roughly, sugar beet pulp only consists of 20–25% weight cellulose [34,35] compared to 50% in wood.

Alkali pre-treatment, which results in increased film density, is also beneficial to the mechanical performance of sugar beet, spruce, and beech. Finally, TEMPO MFC, which is known to provide nanopapers of superior performance [36,37,38], clearly shows the highest tensile strength. Interestingly, the modulus of elasticity of TEMPO MFC nanopaper is matched by alkali-treated fibrillated beech (NaOH beech). Overall, the strength and stiffness of nanopapers produced in the present study compare well with the values found in literature [31]. This also holds true for the elongation at break (Figure 4d). For this parameter, highest values indicating a certain mobility of fibril-fibril bonds were observed for NaOH sugar beet and MFC nanopapers.

**Figure 5:**
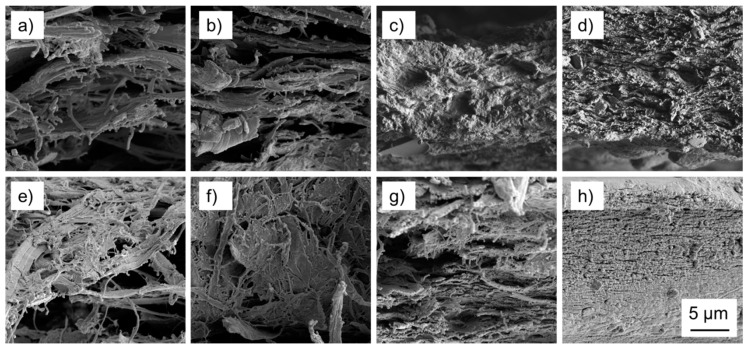
Representative SEM images of fracture surfaces of nanopapers after tensile testing: (**a**) spruce; (**b**) beech; (**c**) sugar beet; (**d**) MFC; (**e**) NaOH spruce; (**f**) NaOH beech; (**g**) NaOH sugar beet; (**h**) TEMPO MFC.

Scanning electron microscopy of the fracture surfaces of nanopapers tested in tension helps understanding some of the differences observed during mechanical characterisation. Clearly, the wood powders directly subjected to fibrillation without any pre-treatment other than swelling in water show insufficient fibrillation (Figure 5a,b). Even though, occasionally, fine individual fibrils are observed, large micron-scale fibrous cell wall fragments dominate the appearance of fracture surfaces. Furthermore, the structure appears rather loose and porous, corresponding well with the low density measured for these variants. Compared to fibrillated wood powder, fibrillated sugar beet pulp (Figure 5c) exhibits a more compact and consolidated surface, and only few individual fibrils may be discerned. Contrary to that, the MFC fracture surface (Figure 5d) is very rough, with many fine fibrils protruding from the material. As to the effect of alkali pre-treatment before fibrillation, only marginal changes are observed for NaOH spruce (Figure 5e), whereas NaOH beech (Figure 5f) shows much finer fibrils compared to untreated beech. Similarly, also the fracture surface of NaOH sugar beet (Figure 5g) shows a much more fibrillary appearance similar to MFC, compared to the untreated variant. The highly fibrillary appearance of fracture surfaces for these two variants agrees well with the hypothesis of inter-fibrillar bond mobility being responsible for their high elongation at break (Figure 4d). On the contrary, TEMPO MFC (Figure 5h) differs from all other variants in that the fracture surface is dense and smooth overall, with many very small fibrils discernible, corresponding with limited elongation at break.

### 3.3. Adhesive Strength by Lap-Shear Testing

**Figure 6:**
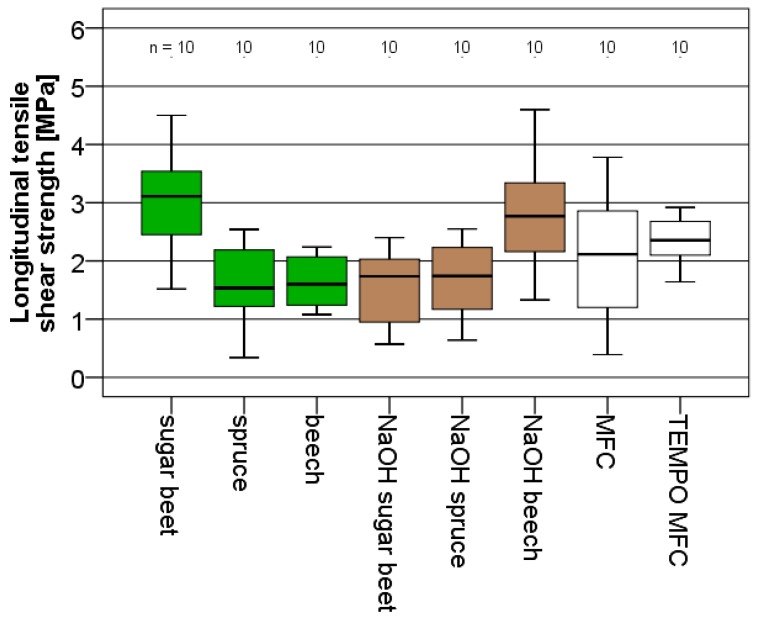
Box and whisker plot of the adhesive strength expressed by shear strength of bonded wood specimens for fibrillated material from different raw materials.

The adhesive strength obtained for different fibrillated materials shown in Figure 6 varied roughly between average values of 1.5 up to 3.0 MPa. Among the tested variants, sugar beet and NaOH beech show best performance. While NaOH beech also performed well in terms of nanopaper strength and modulus, the good performance of untreated fibrillated sugar beet does not correspond to nanopaper performance. Typically, comparable set-ups using spruce wood as a substrate for widely-used commercial wood adhesives, shear strength values in the order of 7 MPa are achieved [39]. Thus, even though substantial self-adhesive (nanopapers) and adhesive strength (lap-shear specimens) is developed, absolute values reach only approx. half the magnitude of standard industrial adhesives.

**Figure 7:**
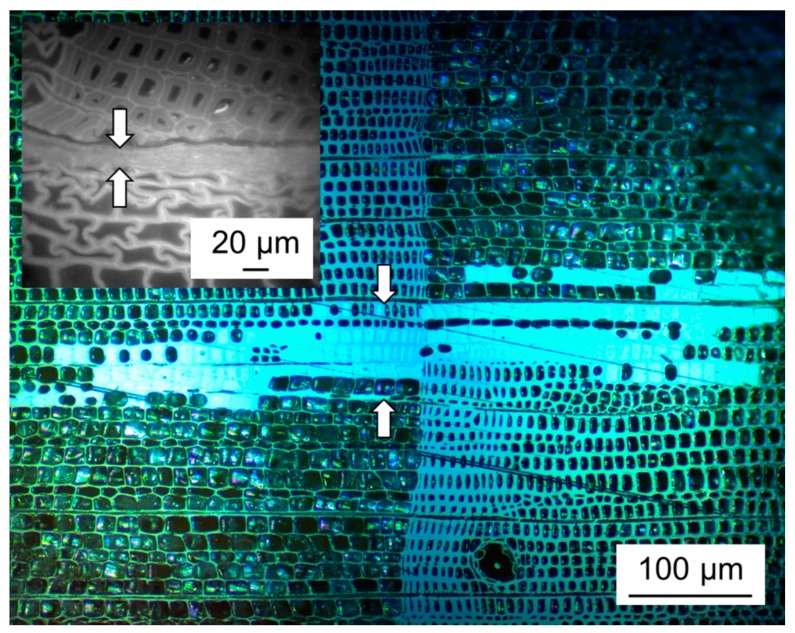
Incident light microscopic image of a typical wood adhesive bond line using the industrial wood adhesive urea-formaldehyde compared to a typical bond line using fibrillated material produced in the present study (inset). Arrows denote the approximate thickness of the bond line.

A discussion of the systems observed in view of adhesion theories may help in understanding the differences observed. According to the state of the art in wood adhesion, polar interactions with wood –OH groups play a dominant role in wood adhesion, while interlocking between the cured adhesive and the porous wood structure may also provide a minor contribution to overall bond strength [40]. As shown in Figure 7, penetration of adhesive into the porous wood structure, which is common for industrial wood adhesives, is not present when using fibrillated (ligno-)cellulose. On the contrary, a very sharp interface between compressed fibrillated material between the bonded wood pieces is discernible for the adhesive bond lines found in the present study. Thus, the potential contribution of this mechanism is lost. Furthermore, penetration of adhesive into wood pores is an indicator of good wetting, which maximises the contact surface between the rough wood substrate and the adhesive used. Lack of penetration into pores is thus clearly a shortcoming of fibrillated (ligno-)cellulose compared to conventional industrial adhesives.

**Figure 8:**
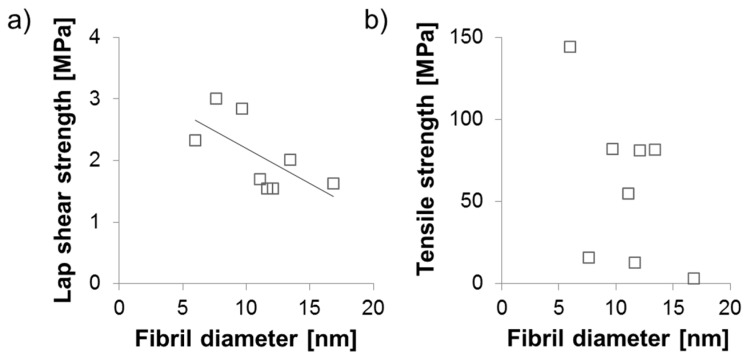
Scatter plots of (**a**) the correlation between fibril diameter and lap shear strength of bonded wood specimens with fibrillated material from different raw materials as well as (**b**) the non-correlating comparison of fibril diameter and tensile strength of nanopapers produced from different raw materials.

With regard to polar interactions, fibrillated (ligno-)cellulose disposes of a high adhesion potential to woody substrate [41], which is facilitated by the intrinsic high specific surface area of this material. With regard to AFM characterisation results shown in Figure 3, it was proposed that the size of the smallest fibrils may be an indicator for differences in the overall specific surface area of the variants studied. A simple correlation analysis of this parameter and nanopaper strength on the one hand, and adhesive strength on the other hand, is shown in Figure 8.

**Figure 9:**
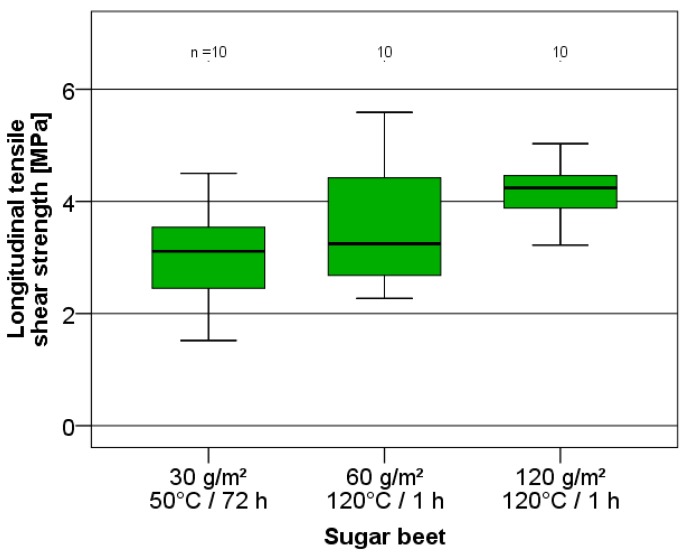
Box and whisker plot of the adhesive strength expressed by shear strength of wood specimens bonded with fibrillated sugar beet pulp using increasing spreading quantities.

While there is no correlation between nanopaper strength and fibril diameter of smallest fibrils, a clear trend of decreasing adhesion strength with increasing diameter of smallest fibrils is apparent. The smallest fibril diameters in ascending order were observed for TEMPO MFC, sugar beet, and NaOH beech (Figure 3). Interestingly, TEMPO MFC, which performs best in terms of nanopaper strength and modulus, shows reduced adhesion strength compared to sugar beet and NaOH beech, respectively. The alkaline treatment of beech wood deacetylates hemicellulose and increases the amount of OH-groups making them more water soluble [42]. This alteration of chemical composition could be a reason for higher adhesive strength because of a better penetration into wood in comparison to TEMPO MFC [43]. It is proposed that particularly in the case of sugar beet, the high content of non-cellulosic carbohydrates may help to explain the good adhesion performance of sugar beet, which shows the best performance of all variants studied. In order to investigate whether further improvement was possible by simply adding more material, the spreading quantity of sugar beet suspension was increased from 30 up to 120 g·m^−2^ (Figure 9). Overall, there is a clear trend of increasing adhesive strength with increasing spreading quantity and strength levels up to more than 60% of the strength level of commercial urea formaldehyde adhesive or similar strengths as starch based wood adhesives [44] are achieved with this fully bio-based variant.

## 4. Conclusions

The results presented above demonstrate that fibrillated material derived from various sources of (ligno-)cellulosic raw materials delivers nanopapers with highly variable properties. Overall, density of the nanopapers formed correlates well with their mechanical performance, with highest densities often also showing highest strength and stiffness, as is well known. Sugar beet, which contains comparatively less cellulose, is an exception to this trend. Regarding adhesive performance of the fibrillated materials, fibrillated sugar beet and fibrillated NaOH pre-treated beech wood perform best, with values up to 60% of the strength of standard industrial adhesives. It is concluded that the adhesion potential of fibrillated (ligno-)cellulose is very promising in dry condition, but is also limited by lack of penetration into the porous wood structure.

## Figures and Tables

**Table 1:** Sample overview with corresponding codes and most relevant chemical treatment.

CODE	Chemical treatment prior to fibrillation
sugar beet	no treatment
spruce	
beech	
MFC	
NaOH sugar beet	NaOH extraction + NaClO_2_ bleaching
NaOH spruce	NaOH extraction
NaOH beech	
TEMPO MFC	oxidation by TEMPO/NaClO/NaClO_2_ system
